# Epigenetic Regulation of Individual Modules of the *immunoglobulin heavy chain locus* 3′ Regulatory Region

**DOI:** 10.3389/fimmu.2014.00163

**Published:** 2014-04-21

**Authors:** Barbara K. Birshtein

**Affiliations:** ^1^Department of Cell Biology, Albert Einstein College of Medicine, Bronx, NY, USA

**Keywords:** *immunoglobulin heavy chain gene locus*, enhancers, insulators, CTCF, Pax5, class switch recombination, somatic hypermutation

## Abstract

The *Igh* locus undergoes an amazing array of DNA rearrangements and modifications during B cell development. During early stages, the variable region gene is constructed from constituent *variable* (*V*), *diversity* (*D*), and *joining* (*J*) segments (*VDJ* joining). B cells that successfully express an antibody can be activated, leading to somatic hypermutation (SHM) focused on the variable region, and class switch recombination (CSR), which substitutes downstream constant region genes for the originally used *C*μ constant region gene. Many investigators, ourselves included, have sought to understand how these processes specifically target the *Igh* locus and avoid other loci and potential deleterious consequences of malignant transformation. Our laboratory has concentrated on a complex regulatory region (RR) that is located downstream of *C*α, the most 3′ of the *Igh* constant region genes. The ~40 kb 3′ RR, which is predicted to serve as a downstream major regulator of the *Igh* locus, contains two distinct segments: an ~28 kb region comprising four enhancers, and an adjacent ~12 kb region containing multiple CTCF and Pax5 binding sites. Analysis of targeted mutations in mice by a number of investigators has concluded that the entire 3′ RR enhancer region is essential for SHM and CSR (but not for VDJ joining) and for high levels of expression of multiple isotypes. The CTCF/Pax5 binding region is a candidate for influencing *VDJ* joining early in B cell development and serving as a potential insulator of the *Igh* locus. Components of the 3′ RR are subject to a variety of epigenetic changes during B cell development, i.e., DNAse I hypersensitivity, histone modifications, and DNA methylation, in association with transcription factor binding. I propose that these changes provide a foundation by which regulatory elements in modules of the 3′ RR function by interacting with each other and with target sequences of the *Igh* locus.

## Discovery of 3′ RR Enhancers

**Figure 1:**
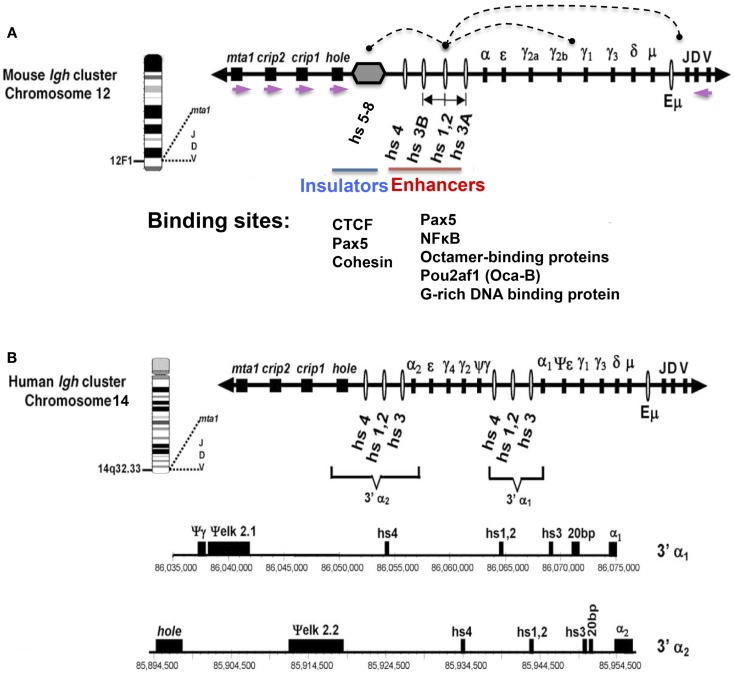
**Schematic representation of mouse (A) and human (B) *Igh* gene loci with emphasis on the 3′ RR**. The depicted orientation reflects the centromere (proximal) to telomere (distal) orientation of the genome sequences. DNase I hypersensitive sites hs3A, hs1.2, hs3B, and hs4 (mouse) (red line); and hs3, hs1.2, and hs4 (human) are enhancers of the 3′ RR. **(A)** Hs1.2 is the center of a palindromic region (double-headed arrow) (see text). The 3′ RR hs5–8 region (gray hexagon) downstream of hs4 contains CTCF sites interspersed with Pax5 sites, and has insulator activity (blue line). Each of the downstream neighboring non-*Igh* genes, *hole* (*Tmem121*), *crip1* and *2*, and *mta-1*, has the same transcriptional orientation (demarcated by purple arrows), which is opposite to that of all the immunoglobulin heavy chain genes. Arcs indicate examples of physical interactions that occur in B cells among the elements of the 3′ RR, and between these elements and regulators of germline transcripts (GT) that are located upstream of switch sequences associated with each *C_H_* gene, or with the expressed *VDJ* gene. Transcription factor binding sites for Pax5, NFκB, octamer-binding proteins, and a G-rich DNA binding protein are present in each of the 3′ RR enhancers (4) (red line). The CTCF-binding region (blue line) has binding sites for Pax5 and cohesin in addition to CTCF (5). **(B)** The human 3′ *Igh* enhancers and other features are shown to scale under the scheme of the locus. Numbers represent the actual location within human chromosome 14 (NT_026437.10). This figure has been used with permission from its original publication in Molecular Immunology. Some annotations and modifications have been added.

The *Igh* locus spans ~3 Mb, beginning near the telomere on murine chromosome 12 with the component *variable* (*V*), *diversity* (*D*), and *joining* (*J*) segments of the variable region, followed by the multiple constant region (*C_H_*) genes (Figure 1). My laboratory has been interested in the regulation of the *Igh* locus’s multiple recombination and mutation processes that generate a diverse antigen-recognition repertoire. The entire 3′ *Igh* regulatory region (RR) (enhancers and insulators) has been shown by others to potentially contribute to regulation of variable region formation (*VDJ* joining) (1). Importantly, it is definitively essential for class switch recombination (CSR) (2), and somatic hypermutation (SHM) (3). This review focuses on our studies on the structure and epigenetic regulation of the 3′ RR as it contributes to those antibody diversification processes.

The first transcriptional enhancer identified in mammalian cells was the intronic enhancer of the *Igh* locus (Eμ), positioned between the 3′-most *J_H_* segment and the 5′-most *C_H_* region, *C*μ [reviewed in Ref. (6)]. Eμ was found to confer expression upon *Igh* genes when transfected into B cells, and was generally considered to be of critical importance in enabling B cell-specific expression of the Igh locus.

Not surprisingly, perhaps, Eμ was not the only B cell-specific enhancer in the *Igh* locus. When B cell lines that had deleted Eμ were found to retain the ability to express the *Igh* gene (7, 8), the questions of gene regulation of *Igh* genes became increasingly provocative. What exactly was Eμ’s role? Was Eμ required to initiate *Igh* expression but not to maintain it? Were there additional enhancers that compensated for the absence of Eμ, and where in the *Igh* locus might they be found? Examining a rat cosmid, the Neuberger group identified a DNA sequence with B cell-specific enhancer activity that was located ~25 kb downstream of *C*α, the most 3′ of the *C_H_* genes (9): this was the first of the 3′ enhancers to be identified.

This newly identified enhancer was satisfyingly predicted to account for the aberrant expression of myc in various B cell malignancies when *myc* was activated as an oncogene via chromosomal translocation with the *Igh* locus. The translocation breakpoints in switch sequences upstream of *C_H_* genes divorced the intronic enhancer from the oncogenic transcription unit, leaving *myc* apparently under the control of the 3′ enhancer [Ref. (9) and reviewed by Vincent-Fabert et al. (10)]. A murine homolog of this enhancer was isolated and named hs1.2 for its two DNAse I hypersensitive (hs) sites (11).

Once more than one *Igh* enhancer was known, i.e., hs1.2 and Eμ, it was natural to ask whether there were additional enhancers. Potential enhancers were identified by DNase I hypersensitivity assays that marked DNA sites that were accessible to transcription factors, and enhancer activity was generally analyzed using transient transfection assays in B cell lines reflecting different stages of B cell development. In a series of experiments by various investigators, using genomic sequences that were only then becoming identified, additional DNase I hypersensitive sites 3′ of Cα were detected, primarily using the mouse locus as a model (12–15). A mouse BAC sequence identified by Roy Riblet was found to encompass the entire 3′ RR and the nearest downstream non-*Igh* genes (AF450245) (16). Similar experiments identified analogous enhancers of the human *Igh* locus (17–19), with the enhancer-containing segment of the human 3′ RR fully characterized by the Max laboratory (20). Figure 1 shows the general features of the enhancer-containing segments of the 3′ RR in both mouse and human. A CTCF/Pax5 binding region with insulator activity is located within the 3′ RR downstream of the 3′ RR enhancers (hs5–8), and will be discussed further below.

## Structural Features of the 3′ RR Enhancer-Containing Regions

There are noteworthy structural features of the 3′ RR (note that this region is sometimes referred to as 3′ Eα) [reviewed in Ref. (21–24) (Figure 1)]. (1) Multiple DNase I HS sites with enhancer activity are dispersed in relatively large DNA segments (~28 kb in mouse) – a total of four enhancers in mouse (hs3a, hs1.2, hs3b, and hs4) and three in humans (hs1.2, hs3, and hs4). (2) In humans, there are two individual 3′ RRs, one each downstream of *C*α*1* and *C*α*2*, respectively. They are quite similar to each other in sequence. The orientation of hs1.2 with respect to upstream and downstream sequences is reversed in the two 3′ RRs in human, and also between rat and mouse 3′ RRs (18). (3) A conserved palindrome feature, although not its specific sequences, flanks the central enhancer – hs1.2 (25). In mouse, the palindrome extends in both directions from hs1.2 to terminate at two virtually identical enhancers, hs3a and hs3b (26). Compared to mouse, the hs1.2 palindromic region in humans is shorter (27). Other species also have a conserved palindrome (28). (4) The hs4 enhancer is located outside and downstream of the palindrome. (5) Individual 3′ RR enhancers in a given species, like hs1.2, hs3, and hs4, differ in sequence from each other (except for the virtually identical hs3a and hs3b in rodents). (6) There are limited homologies in enhancer sequence between species (e.g., hs1.2 in human and hs1.2 in mouse) (27).

Other than revealing a conserved palindromic structure, the regions between the 3′ RR enhancers show virtually no homology between rodents and humans (27). Nonetheless, other particular sequence features stand out, as identified through genomic Southern analysis and percentage identity (dot-plot) analysis (29). In mouse (and rat), the “palindromic” sequences that separate hs1.2 from each of the terminal enhancers at the end of the palindrome, hs3a and hs3b, contain families of direct and inverted repeats (26) while the human (and chimpanzee) 3′RR revealed several regions of repetitive switch-like sequences (27). More recently, the Cogne laboratory specifically sought and identified multiple switch-gamma 1-like repeats in the mouse and human 3′ RRs that were situated close to each of the four enhancers, as well as less distinct although evident, similar sequences in other species, like rabbit and dog (30). In mouse, the 3′ RR is highly polymorphic (26, 31), showing variations in the lengths of the sequences between the enhancers and the number of repeats in these regions. The hs1.2 region in humans is polymorphic, with varying frequency of alleles in different populations (32). Polymorphic patterns of human hs1.2, i.e., alleles, are associated with different autoimmune disorders, such as lupus (33).

In summary, the 3′ RR contains several enhancers located in two structurally distinctive modules – (1) a palindromic region (mouse hs3a–hs1.2–hs3b) and (2) a separate structural unit (hs4). Inter-enhancer regions reveal repetitive, switch-like sequences potentially of functional significance for the Igh locus. Downstream of the enhancer-containing segment of the 3′ RR is additional DNase I hypersensitive sites (hs5–8), which contain CTCF and Pax5 sites and have insulator activity. This hs5–8 region is discussed more fully later.

## Transcriptional Regulation of 3′ RR Enhancers

Relatively coincident with these studies on the *Igh* locus were studies of genes of the β-*globin* locus, which, like the *Igh* genes, are subject to developmental regulation. Multiple DNase I hypersensitive sites, each with enhancer activity, are located upstream of the β-*globin* genes. This enhancer-containing region is referred to as the locus control region because endogenous deletions here are found to affect expression of distally situated globin genes. Experimental questions on *Igh* genes have paralleled experiments carried out in the β-*globin* locus and in other loci [recent review in Ref. (34)]: (1) what are the protein factors that bind to and regulate these *Igh* enhancers? Can they account for B cell-specific regulation? (2) Are the different enhancers similar in their function, their relative “strength” and their activity on the target *Igh* locus? How do these enhancers work together? Can these questions be answered not only for *in vitro*, cellular conditions but also within the animal context itself?

Electrophoretic mobility shift assays (EMSA) provided a tool to identify proteins with the potential to bind enhancers. Using nuclear extracts, we identified a B cell-specific binding protein with sites throughout the *Igh* locus, including hs1.2 (35, 36). Based on its cellular expression pattern, we predicted that this protein was B cell-specific activating protein (BSAP), now called Pax5, as originally identified by the Busslinger laboratory (37); and various observations were consistent with that prediction (36). Additional 3′ RR binding factors were identified leading to recognition of a quartet of proteins – Pax5, octamer-binding proteins, NFκB, and a G-rich DNA binding protein – that worked together on each of the murine 3′ enhancers (4) (Figure 1).

Our experiments revealed that BSAP/Pax5 bound to each of the mouse 3′ RR enhancers, where it could act as a repressor or activator (4). For example, mutational inactivation of a BSAP/Pax5 binding site of hs1.2 resulted in an *increase* in hs1.2 enhancer activity upon transfection into B cell lines that expressed endogenous BSAP/Pax5 (36). This finding showed that “BSAP” could be a repressor of hs1.2. The enhancer activity resulting from mutation of the BSAP/Pax5 binding site depended on the binding of the remaining transcription factors (38). A similar outcome applied not only to BSAP/Pax5 but also to each individual component of this quartet, as individual mutation of other binding sites each resulted in an increase in hs1.2 enhancer activity (38). Collectively, then, this quartet worked in concert to repress hs1.2, while it activated hs4, revealing that individual 3′ RR enhancers had different B cell-specific activities (4). Interestingly, human 3′ enhancers do not have Pax5 binding sites, suggesting that humans and mice have different modes of 3′ RR regulation. However, it is not known how or whether the differences in Pax5 binding affect the function of the 3′ RR in human and mouse. Human hs4 showed binding to octamer-binding proteins, NFκB, and YY1 under some circumstances, and human hs1.2 to octamer-binding proteins and Spi1, and to NFκB for some of the polymorphic hs1.2 variants (33, 39–41). Other 3′ enhancer binding proteins have also been identified (40, 42).

## Additional Module Downstream of 3′ RR Enhancers: The CTCF-Binding Region of the 3′ RR

Had we identified all the regulators of the 3′ RR [reviewed in Ref. (43)]? Various observations suggested that additional functional motifs were present beyond hs4. For example, the nearest non-*Igh* genes downstream of hs4, i.e., *hole* (*Tmem121*), *Crip*, and *mta-1*, each had a transcriptional orientation that was opposite to that of all of the *V*, *D, J*, and *C_H_* elements of the *Igh* locus. This back-to-back orientation led us to predict that a terminus of the *Igh* locus might be located in this segment (16). In fact, we found additional DNase I hypersensitive sites downstream of hs4, which included hs5, 6, and 7, and has now been extended to include a CTCF-binding site, named hs8 (44, 45). Discussions via Sandy Morse with Victor Lobanenkov introduced us to CTCF as a mammalian insulator (46), and we predicted that CTCF sites might be present in this region. EMSA of 50 overlapping DNA sequences with recombinant CTCF revealed a CTCF-binding module of the 3′ RR [recently referred to as 3′ CBE, CTCF-binding elements (47)], and transient transfection assays confirmed functional insulator activity in the absence of any enhancer activity (44). The CTCF sites are interspersed with Pax5 binding sites within the hs5–8 region (5).

## Functional Analysis of 3′ RR Regulatory Elements through Targeted Deletions

### 3′ RR enhancers

Analysis of the function of the endogenous 3′ RR began with the description of spontaneous 3′ RR deletion mutants identified in cell lines. For example, a low-producing variant (LP1.2) of a mouse plasmacytoma cell line was shown to have sustained a deletion of the entire 3′ RR (15, 48). This suggested that the 3′ RR supported high levels of *Igh* expression in plasma cells. With the development of both transgenic (49, 50) and endogenous models, the 3′ RR has been over many years the focus of multiple targeted deletions [reviewed in Ref. (24)]. Although the efficiency of targeting of this 3′ RR region has been hampered, perhaps because of its complex structure, there has been gradual, ongoing success. Deletion of individual enhancers had no significant phenotypic consequence implying that the remaining elements, each constellation of which is different, can provide 3′ RR function. Deletion of two or more enhancers gave phenotypic consequences of varying degrees, e.g., deletion of hs3b and hs4 together eliminated class switching to all isotypes except for IgG1 (51). Now, there are mice from which the entire ~28 kb 3′ enhancer region has been deleted (2), and these have provided a clear demonstration of the potency of the complete 3′ RR enhancer region. Without 3′ RR enhancers, mice are able to express only reduced levels of IgM at the plasma cell stage, they lack class switch recombination to all isotypes (2) and they are deficient in SHM (3). There is no impairment of *VDJ* joining (52). Studies by the Cogne laboratory showed that 3′ RR enhancers hs1.2 and hs4 were transcriptionally active in B cells, and hs1.2 could be targeted by AID, revealed by detectable although relatively low levels of SHM (30). These AID-dependent mutational and recombination processes involving the 3′ RR with Sμ resulted in deletion of the entire *IgC_H_* region and B cell death (30). This revealed an ongoing competition between generation of live class switched mutated B cells and dead B cells, termed “locus suicide” by the authors. In all, these data strongly show that the 3′ RR enhancer region (hs3a–hs4) is critical for CSR and SHM and functions through synergy among the multiple 3′ RR enhancers.

### CTCF/Pax5 binding region

Similar to the analysis of the 3′ RR enhancers, we used targeted deletion to examine the effect of the CTCF/Pax5 binding region of the 3′ RR on *Igh* expression (53). We were surprised to find that deletion of the 8 kb hs5–7 region resulted only in a mild phenotype. There was an increase in recombination of the most proximal *D* gene, *DQ52*, to *J_H_3*, a reduction in contraction between distal *V_H_J558* and proximal *V_H_7183* genes, and an ~2-fold increase in *V_H_7183* gene usage-all suggesting a modest contribution of the CTCF/Pax5 region of the 3′ RR to steps in VDJ joining. Nonetheless, upon targeted deletion of hs5–7, there were essentially normal levels of *Igh* recombination for *V_H_* formation and CSR, normal levels of *Igh* expression and allelic exclusion, and B cell development was unaffected. One possibility to account for these observations was our finding that two CTCF sites remained downstream of the seven sites that had been deleted, in the segment called “38” in the manuscript and now termed hs8. In addition, CTCF sites are associated with each of the downstream non-*Igh* genes. This suggests that a full deletion of CTCF sites in this region might reveal a more extensive phenotype.

## Physical Interaction of the 3′ RR with Its Target Sites in the *Igh* Locus

*V_H_* promoters and *I* promoters that drive germline transcription (GT) for CSR are situated quite far in a linear distance from the 3′ RR; yet it is implied that they all function together through physical interaction (Figure 1). In fact, our finding of an inversion of the *Igh* locus in a variant of the MPC11 plasma cell line that extended from the *V_H_* through to the 3′ RR (54) was indicative of a loop formed by interactions between DNA sequences at *V_H_* and 3′ RR inversion breakpoints (55). Chromosome conformation capture (3C) technology has been important in documenting interactions that occur in a cellular context, by fixing these by formaldehyde cross-linking, cutting away intervening DNA stretches with restriction enzymes, ligating remaining neighboring fragments, and documenting these interactions by PCR with selected primer pairs. Using 3C, we sought to confirm the dependence of H chain expression in plasma cells on an intact 3′ RR (55): indeed, we found that the 3′ RR interacted with the *J_H_*–Eμ region. This interaction took place even in cells in which Eμ was deleted. Not only was there interaction between the 3′ RR and its target sequences, but there was also interaction among component 3′ RR enhancers and insulators, including the CTCF/Pax5 binding unit (hs5–8) (3′ CBE). Notably, substitution of hs1.2 by the *NeoR* gene in a variant of the MPC11 plasma cell line resulted in loss of *Igh* expression (56) and abrogation of the 3′ RR loop structure; i.e., looping was essential for *Igh* expression. Collectively, these experiments show that the entire 3′ RR, including enhancers and insulators, works as a physical unit.

The Kenter laboratory focused on normal spleen cells stimulated to undergo switching for their 3C experiments (57). They reported that in resting B cells, but not in T cells, the 3′ RR interacted with the *VDJ*–Eμ region. Upon LPS ± IL4 stimulation of splenic B cells, they found that specific *I/switch* regions that drive GT were brought into the *VDJ*–3′ RR loop. Splenic B cells from mice that were unable to carry out GT and CSR as a result of the combined deletion of the hs3b and hs4 3′ RR enhancers failed to show these interactions.

Interestingly, mice bearing the combined deletion of hs3a and hs3b (58) had no defects in GT or CSR, but interactions between the 3′ RR and *I/switch* regions that ordinarily were cytokine-dependent were already at an induced level in the hs3a/hs3b deleted mouse. Collectively, these data provided support for a loop interaction model by which H chain expression and CSR are dependent on physical interaction of the 3′ RR with target *Igh* sequences.

Presuming that the CTCF/Pax5 region (3′ CBE) of the 3′ RR interacts with other *Igh*-associated CTCF sites, such candidate CTCF target sites have been defined by colleagues using array analysis and genome-wide ChIP (59, 60). Moving 3′–5′ upstream of the 3′ RR, there are no CTCF sites in *C_H_* and *J_H_* genes until those detected in the 5′ *D_H_* segment (1, 61). The *V_H_* region contains multiple CTCF sites, some associated with specific Pax5 binding sites, termed PAIR (62). Recent studies showed that the 3′ RR CTCF/Pax5 binding region interacts with the two *D_H_*-associated CTCF sites (IGCR1), targeted deletion of which showed their critical role in appropriate regulation of VDJ joining (1). We might, therefore, predict that the deletion of the complete 3′ RR CTCF-binding region with which IGCR1 interacts would have a major influence on VDJ joining.

Genome-wide analyses have been used to identify interactions between 3′ RR elements, e.g., hs3b and hs8, and the rest of the *Igh* locus (47, 63). Studies with 4C (47) have identified Pax5-dependent interactions in Rag^−/−^ pro-B cells where *V_H_* genes are poised to contract prior to VDJ joining. These 3′ RR interactions are maintained even when individual regulatory elements, such as Eμ, IGCR1, and the entire 3′ RR enhancer region from hs3A to hs4, are deleted. This implies some independent means of interaction, perhaps involving retained 3′ RR CTCF-binding sites, or synergy among regulatory elements that enables continued interactions even when single elements are deleted. Notably, chromatin interaction analysis by paired-end tag sequencing (ChIA-PET) of long-range chromatin interactions has revealed interactions of the 3′ RR with transcribed *Igh* genes in B cells activated by LPS + IL4 that are not detected in embryonic stem (ES) cells (63), in accord with developmental differences in 3′ RR interactions.

## What Proteins Support Loop Formation Involving the 3′ RR?

To tackle this question, we used lentiviral delivery of shRNA directed against expression of CTCF, Oct-2, and a coactivator of Oct-2, namely Pou2af1 [i.e., OCAB, OBF-1, BOB1, each of which, as proteins that bound to the 3′ RR, was a candidate for loop promotion (64)]. Despite reduced levels of these proteins in response to shRNA, no alterations in loop formation or *Igh* expression were observed in the mouse MPC11 plasmacytoma cell line we examined. Interestingly, in contrast to our observations, there was a report that reduction of *Pou2af1* expression in the same plasma cell line using step-wise selection of a cell line containing two independent shRNA’s led to reduction in *Igh* expression and 3C interactions (65). Accordingly, we concluded (64) that there are likely some conditions under which Pou2af1 can facilitate 3C interactions involving the 3′ RR, among them the possibility that this approach had selected a variant cell line that was dependent on Pou2af1. Potentially, under other conditions, 3C interactions depend on a protein other than Pou2af1, or on synergy involving more than one protein, or on the low levels of individual proteins remaining from inefficient knock down.

## Epigenetic Regulation of 3′ RR during B Cell Development

Over several years, we have worked to know how components of the 3′ RR are individually regulated, enabling them, in turn, to act together as a unit in CSR and SHM, and potentially also for VDJ joining. “Active” DNA segments are generally associated with DNase I hypersensitivity, specific histone marks, and DNA demethylation, which will be discussed individually below.

### Histone marks

Non-B cells that were studied had varying profiles of histone acetylation of the 3′ RR (66). For example, a macrophage cell line had active marks of AcH3 and AcH4, while T cells lacked both AcH3 and AcH4. In mouse erythroleukemic (MEL) cells, the CTCF-binding region, but not 3′ RR enhancers, was associated with acetylated histones. Therefore, active histone marks of the 3′ RR were not necessarily B cell-specific. In B cells, modules of the 3′ RR sequentially acquire active histone marks during development (44). The CTCF-rich region first acquires these marks, followed progressively 5′ to hs4 and then to the palindromic enhancers. ChIP experiments of the 3′ RR showed that in pro-B cells, hs5 and hs6 of the CTCF-binding region were associated with AcH4 and low levels of AcH3, while hs4 was also associated with AcH4 but not with AcH3. In pre-B cells, the entire hs4–8 region was associated with both acetylated marks; and then in B cells, the palindromic enhancers also acquired these marks. These observations suggest step-wise activation of different modules of the 3′ RR during B cell development, raising the possibility that specific combinations of 3′ RR modules, involving palindromic enhancers, hs4, and the CTCF-binding region, have specific functional contributions.

### DNA demethylation

Early studies had bluntly monitored DNAse I hypersensitivity and DNA demethylation in the region now shown to contain the entire 3′ RR (12), but as the complete 3′ RR structure became known, a finer analysis was made possible (66). The CTCF-binding region was generally constitutively demethylated in all cell types analyzed. In several sources of non-B cells, the 3′ RR’s palindromic region was demethylated without a corresponding association with active histone marks. However, in B cells, three epigenetic marks – DNase I hypersensitivity, active histone marks, and DNA demethylation – were collectively engaged; and progressive demethylation paralleled acquisition of active histone marks. Hs4 and downstream CTCF-binding sites were DNase I hypersensitive and demethylated, as assessed by relative sensitivity to the methylation-sensitive isoschizomers *Hpa*II and *Msp*I, beginning in pro-B cells and extending throughout B cell development. The palindromic region became hypersensitive and partially demethylated only later in B cell development. We found upon comparison of wild-type with Pax5-deficient pro-B cells in which Pax5 expression could be reinitiated, that in the absence of Pax5, there was scattered demethylation of the palindromic region. Re-expression of Pax5 could promote methylation of the palindromic region. These findings suggested that Pax5 was a critical factor in over-all B cell-specific epigenetic regulation of the 3′ RR. In other studies involving targeted deletion of linker histone *H1* genes, we found that linker histone H1 was also important for the methylation of hs4–hs8 in wild-type ES cells.

## Epigenetic Regulation of 3′ RR during CSR

Despite the critical role of the 3′ RR in CSR (and SHM), there are no apparent changes in histone marks in the 3′ RR during switching in cultured cells (44). This implies that the 3′ RR in resting B cells is already epigenetically poised for its activity for CSR. Instead, we have observed dynamic changes in Pax5 interaction over time in response to LPS stimulation (5). In resting B cells, Pax5 bound hs4 and the 3′ RR was mostly methylated. When GT was at a peak at ~48 h after commencement of LPS stimulation, Pax5 binding had shifted from hs4 upstream to hs1.2 and downstream to hs7. By 72 h, when CSR was essentially complete, Pax5 had resumed its beginning position at hs4. ChIP analysis of cell sources that were deficient in GT or in CSR showed differences in these Pax5 binding patterns, in accord with the notion that shifts in Pax5 binding reflected mechanisms by which the 3′ RR supported GT and CSR. We have proposed a model by which mouse 3′ RR enhancers form a scaffold through which Pax5 can interact. Deletion of any individual enhancer leaves residual Pax5 sites in each of the remaining enhancers and in the CTCF/Pax5 binding region, which allows the 3′ RR to remain functional.

## On the Horizon: Experiments on the 3′ RR

### How does the 3′ RR function?

The 3′ RR enhancer region is critical for GT and CSR, and SHM, and the CTCF/Pax5 binding region could contribute to VDJ joining. We predict that a scaffold formed by modules of the 3′ RR supports physical interactions with target sequences required to accomplish these various activities. With deletions of individual 3′ RR enhancers having little phenotypic consequence, one can ask how many different structural solutions are there to 3′ RR activity? Why are there multiple modules? Do the changes in epigenetic alterations of 3′ RR modules that occur during development indicate specific activities for individual modules? Could the 3′ RR help target DNA repair proteins involved in SHM or CSR? What roles does the 3′ RR share with other cis acting sequences that are critical for SHM, such as those in the light chain loci? What is distinctive about CSR, which is specific to the *Igh* locus? What is the role of the conserved palindrome? How did the 3′ RR evolve? What are the species-specific aspects of 3′ RR regulation?

Recent experiments on the β-*globin* LCR have identified hierarchical regulation by multiple transcription factors (67). Binding of individual factors can provide a foundation for subsequent binding of other factors. Experiments of these types on the 3′ RR could be equally informative in answering how this region functions. Indeed, more complete deletion of 3′ RR CTCF-binding sites, and targeted deletions and mutations in 3′ RR modules would also be informative. The new CRISPR technology (68) should facilitate these constructions and provide answers to many questions.

### Is the CTCF/Pax5 binding region the terminus of B cell-specific regulators of the *Igh* locus?

A persuasive set of experiments says “yes” to the role of the 3′ RR as a terminus of *Igh* regulation via chromatin accessible marks. These experiments have shown that active chromatin marks extend unilaterally 3′–5′ from the 3′ RR as far as 450 kb when *Igh-myc* translocations are assayed in endemic Burkitt lymphoma samples (69). This supports the identification of the CTCF/Pax5 region as a functional insulator of the Igh locus. In addition, 4C studies have implicated hs8 as a 3′ boundary for *Igh* locus interactions (47). Yet, as described below, there is a replicative terminus further downstream, raising the possibility of additional *Igh* locus regulators.

### Role of replicative terminus downstream of CTCF/Pax5 region

Our experiments in collaboration with the Schildkraut laboratory identified an origin of an ~500 kb *Igh* temporal replicative transition region in MEL (non-B) cells. DNA replication initiates ~45 kb downstream of the CTCF/Pax5 module of the 3′ RR between *crip1* and *Tmem121* (Figure 1) and extends progressively 3′–5′ throughout S phase to replicate the 3′ RR, *C_H_, J_H_, D_H_*, and most proximal *V* regions (70, 71). All *V_H_* genes replicate late in S phase. In pre-B cells, the entire *Igh* locus replicates early in S phase, indicating the firing of multiple origins that are ordinarily quiescent in non-B cells (71). In B cells, a temporal transition region is again apparent, but origins appear to be closer to or within the 3′ RR, suggesting that the replication landmark is flexible (72). These data implied that *Igh* replication is under B cell-specific developmental control. In MEL cells, the downstream origin, which is located beyond the limits of the 3′ RR, may be a terminus for *Igh* locus regulation. It is of interest that changes in *Igh* DNA replication are associated with changes in nuclear location of the *Igh* locus (71, 73) but can be independently regulated (74). In pro- and pre-B cells, the *Igh* locus is located away from the nuclear periphery, while in MEL and ES cells, and in B and plasma cells, the *Igh* locus is located at the nuclear periphery. These observations raise the question of whether there are finer demarcations of nuclear subcompartments generally associated with the *Igh* locus and the 3′ RR? What regulates the movement of the locus from one position to another?

### Is the 3′ RR involved in inter-chromosomal interactions?

The mechanism by which recurrent translocations involving the *Igh* locus take place and the role of the 3′ RR are under close scrutiny (75, 76). Epner and colleagues have reported a role for the 3′ RR in transvection involving allelic interactions (77). Further, our studies have identified a region between hs4 and hs5 that has a methylation signature indicative of allelic expression (66). The Skok laboratory has observed allelic interactions in *Igh* genes, which are evident during steps of VDJ joining (78). The various 3C technologies and their broader counterparts, as noted in part above (47, 63), should be very informative about the contribution of the 3′ RR to genetic domains of interaction.

### Is there a role of the 3′ RR as a super enhancer?

Recent genome-wide studies have reported “super enhancers” (79, 80), DNA segments substantially larger than other “enhancer” regions and identified as having strong binding sites for BRD4, a member of the bromodomain and extraterminal (BET) subfamily of human bromodomain proteins, and for the Mediator complex with which BRD4 interacts. By these criteria, the 3′ RR was predicted to be a super enhancer in multiple myeloma cells (79), where it upregulates expression of the *myc* oncogene to which it is juxtaposed as a result of a chromosomal translocation. An inhibitor of BRD4, JQ1, can lead to downregulation of *myc* expression in multiple myeloma cells. However, *myc* appears to be suppressed by JQ1 regardless of whether it is associated with *Igh* sequences through translocation (81), potentially via B cell-specific enhancers of *myc* (47, 63). Is the 3′ RR a super enhancer? Under what circumstances? Does the 3′ RR share features in common with other “super enhancers”? How might the 3′ RR become a super enhancer?

## Conflict of Interest Statement

The author declares that the research was conducted in the absence of any commercial or financial relationships that could be construed as a potential conflict of interest.
